# Factors associated with dietary diversity and length‐for‐age *z*‐score in rural Ethiopian children aged 6–23 months: A novel approach to the analysis of baseline data from the Sustainable Undernutrition Reduction in Ethiopia evaluation

**DOI:** 10.1111/mcn.12852

**Published:** 2019-07-13

**Authors:** Desalegn Kuche, Cami Moss, Solomon Eshetu, Girmay Ayana, Mihretab Salasibew, Alan D. Dangour, Elizabeth Allen

**Affiliations:** ^1^ Food Science and Nutrition Research Directorate Ethiopian Public Health Institute Addis Ababa Ethiopia; ^2^ London School of Hygiene and Tropical Medicine London UK

**Keywords:** causal diagram, dietary diversity, length‐for‐age, nutrition

## Abstract

Infants and young children need diets high in nutrient density and diversity to meet the requirements of rapid growth and development. Our aim was to evaluate sociodemographic, agricultural diversity, and women's empowerment factors associated with child dietary diversity and length‐for‐age *z*‐score (LAZ) in children 6–23 months using data collected as part of the Sustainable Undernutrition Reduction in Ethiopia (SURE) evaluation study baseline survey in May–June 2016. We here present a novel analysis using directed acyclic graphs (DAGs) to represent our assumptions about the causal influences between the factors of interest and the outcomes. The causal diagrams enabled the identification of variables to be included in multivariable analysis to estimate the total effects of factors of interest using ordinal logistic/linear regression models. We found that child dietary diversity was positively associated with LAZ with children consuming 4 or more food groups having on average an LAZ score 0.42 (95% CI [0.08, 0.77]) higher than those consuming no complementary foods. Household production of fruits and vegetables was associated with both increased child dietary diversity (adjusted *OR* 1.16; 95% CI [1.09, 1.24]) and LAZ (adjusted mean difference 0.05; 95% CI [0.005, 0.10]). Other factors positively associated with child dietary diversity included age in months, socio‐economic status, maternal education, women's empowerment and dietary diversity, paternal childcare support, household food security, fruit and vegetable cultivation, and land ownership. LAZ was positively associated with age, socio‐economic status, maternal education, fruit and vegetable production, and land ownership.

1

**Key messages**

- Young children in Ethiopia are slow to begin consuming a range of diverse complementary foods.
- Child dietary diversity is positively associated with linear growth; and household production of fruits and vegetables is positively associated with child dietary diversity and length‐for‐age *z*‐score.
- Gender‐sensitive interventions to increase maternal education, women's empowerment, and paternal support for childcare may support improved diversity of child feeding.

## 2 INTRODUCTION

Infants and young children require diets high in nutrient density and variety to meet the requirements of rapid growth and development (Black et al., 2013). Growth failure is most pronounced between ages 6 to 23 months of age (Shrimpton et al., 2001) and stunting—or low length‐for‐age *z*‐score (LAZ)—is associated with poorer cognitive development, weakened immune systems, and increased risk of chronic disease later in life (Dewey & Begum, 2011). Globally, only 29% of children 6–23 months consume the minimum dietary diversity (defined by WHO as ≥4 of 7 food groups; UNICEF, 2016) and an estimated 23% of children under 5 are stunted (UNICEF, 2017).

Dietary diversity is associated with stunting in multiple low‐income settings characterised by high prevalence of undernutrition (Arimond & Ruel, 2004). In Ethiopia, children's diets are among the least diverse in the world (12.5% of children 6–23 months consume the minimum dietary diversity; UNICEF, 2018) and 37% of children under 5 are stunted (Central Statistical Agency, 2016). The Federal Government of Ethiopia has committed to improving the nutritional status of children, developing the third National Nutrition Programme (2016–2020) to drive policy actions across multiple key sectors including health, agriculture, education, and business (Federal Democratic Republic of Ethiopia, 2016). Infant and young child feeding (IYCF) practices are highlighted and the plan calls for the cross‐sectoral delivery of nutrition‐sensitive interventions to improve child dietary diversity and, consequently, reduce stunting.

In support of these objectives, the government designed the Sustainable Undernutrition Reduction in Ethiopia (SURE) programme, a multisectoral intervention that aims to integrate the work of the health and agriculture sectors to improve child feeding and reduce stunting. Household counselling and participatory community events are delivered by health and agriculture extension workers to improve IYCF and nutrition‐sensitive agriculture practices; and multisectoral governance structures are supported at district and *kebele* (subdistrict) levels. Since June 2017, SURE has been implemented in 50 districts in the four agrarian regions of Ethiopia (Amhara, Oromia, Tigray, and SNNP) and reaches approximately 800,000 children aged 6 to 23 months.

Risk factors for low LAZ or stunting have been studied in settings around the world including Ethiopia (Danaei et al., 2016), but context‐specific risk factors for dietary diversity are less understood as is the contribution of dietary diversity to linear growth. In particular, agricultural diversity and women's empowerment—factors central to the SURE programme evaluation—and their respective relationships to child diets and growth have been less researched and thus comprise a particular focus of this research. Using data from the baseline survey, this study uses novel methods—construction of directed acyclic graphs (DAGs) to diagram causal relationships—to evaluate the association between child dietary diversity and LAZ in agrarian regions of Ethiopia and to identify risk factors for each of the two nutrition‐orientated outcomes.

## 3 METHODS

### 3.1 Study design

Evaluation of the SURE intervention uses a quasi‐experimental study design to determine the impact on child minimum acceptable diet (MAD) and stunting. This study analyses data from the SURE baseline survey completed in May–July 2016 and comprising 1,848 children 6–23 months of age in 36 intervention districts (Oromia: *n* = 18; Amhara: *n* = 8; Tigray: *n* = 4; and SNNP: *n* = 6) and 36 comparison districts selected by region in equal proportion. Complete details are available in the SURE evaluation study protocol (Moss et al., 2018).

### 3.2 Sampling

At baseline 4,980 children 0–47 months (761 children 0–5 months; 1,848 children 6–23 months; 2,371 children 24–47 months) were selected from 4,299 households. Sample size calculations were based on detecting a change at endline in LAZ/height‐for‐age *z*‐score (HAZ) score and MAD attributable to the intervention. Detectable differences of 0.15 HAZ and of 4% MAD were calculated for intracluster correlation coefficients of 0.03 with 80% power with a significance level of 5%; and differences of 0.21 HAZ and 6% MAD were calculated for intracluster correlation coefficients of 0.08 with 80% power with a significance level of 5%.

*Kebeles* were selected at study outset using probability proportional to size sampling from lists and population data provided by district officials. *Gotes* (sub‐*kebeles*) were selected by simple random sampling (paper in hat) during data collection. A complete listing of all households with children under 47 months in the *gote* was conducted, and 15 were selected using systematic random sampling. Resident children 0–47 months within a selected household were listed in the following age groups: 0–5 months, 6–23 months, and 24–47 months. Where only one child for any or all age categories was present, all eligible children were selected (up to three children). Where multiple children from a single age category were present, one child was randomly selected per age group by the computer‐assisted personal interview programme. This study uses data from children sampled who were between 6 and 23 months of age only as this is the age group for which WHO complementary feeding indicators apply and for whom dietary data were collected.

### 3.3 Questionnaire and anthropometry

The household questionnaire (see Data S1) comprised modules on child feeding and care practices, child anthropometry and haemoglobin, household characteristics including food security, mother's dietary diversity, agricultural practices including diversity of food production, and women's empowerment. Child anthropometric measurements comprising length were taken using a portable measuring board (UNICEF Supply Division, Copenhagen, 2016). Data collectors were trained in anthropometric measurement for 5 days and completed a standardisation exercise prior to survey deployment. Survey training on the questionnaire, operating procedures, and piloting was completed in 12 days for a total of 17 days of training.

### 3.4 Data management

#### 3.4.1 Length‐for‐age

Child LAZs were generated using the WHO growth standards (WHO Multicentre Growth Reference Study Group & de Onis, 2006). Scores of <−6 or >6 LAZ were excluded for biological implausibility as per WHO guidelines (World Health Organisation, 2006).

#### 3.4.2 Dietary data and food insecurity

Dietary diversity was first generated for children as a score ranging from 0 to 7 food groups as defined by WHO indicators for IYCF (WHO et al., 2010). We then combined 4–7 food groups consumed into a single category to create a five‐category child dietary diversity outcome variable: 0, 1, 2, 3, and 4–7 food groups consumed. Women's minimum dietary diversity was generated as a scale variable ranging from 0 to 10 food groups as defined by FAO/FANTA (Food and Agriculture Organisation & USAID's Food and Nutrition Technical Assistance III Project (FANTA), 2016). A scale variable from 0 to 27 based on the Household Food Insecurity Access Score was also used (Coates, Swindale, & Bilinsky, 2007). All dietary data were cleaned by comparing 24‐hr recall foods first entered in computer‐assisted personal interview questionnaire as free text with final food group assignments made by data collectors.

#### 3.4.3 Sociodemographic variables

A household wealth index was created using principle components analysis applied to proxy indicators of household socio‐economic status, namely, ownership of consumer goods, electricity, livestock (non‐food producing), source of water, type of toilet, and type of materials used for floor, roof, and walls. We created tertiles and checked internal validity by assessing ownership of consumer goods and housing characteristics by socio‐economic status tertile. Maternal education, land ownership, and ownership of livestock producing animal sources foods such as cows or sheep were excluded from the index due to known effects on nutrition outcomes that we wished to explore independently.

#### 3.4.4 Agricultural production

Variables for household food production were constructed from crops grown, animals reared, and resulting food types produced by the household within the past major and minor growing seasons (1‐year reference period). We constructed a variable for fruit and vegetable production as the sum of all distinct types of fruit and vegetable crops grown in the past year. The score ranged from 0 (i.e., no fruit and vegetable crops grown) to a maximum of 21 as reported by the household. A variable for animal source food production was constructed as the sum of all such food types produced on a scale of 0–10 including eggs, meats, milks and other dairy, and fish.

### 3.5 Statistical analyses

Descriptive statistics were generated for child, mother, and household risk factors and outcomes. Continuous variables were summarised using means and standard deviations or medians and interquartile ranges (IQRs) for nonnormally distributed variables. Categorical variables were summarised using numbers and percentages.

**Figure 1:**
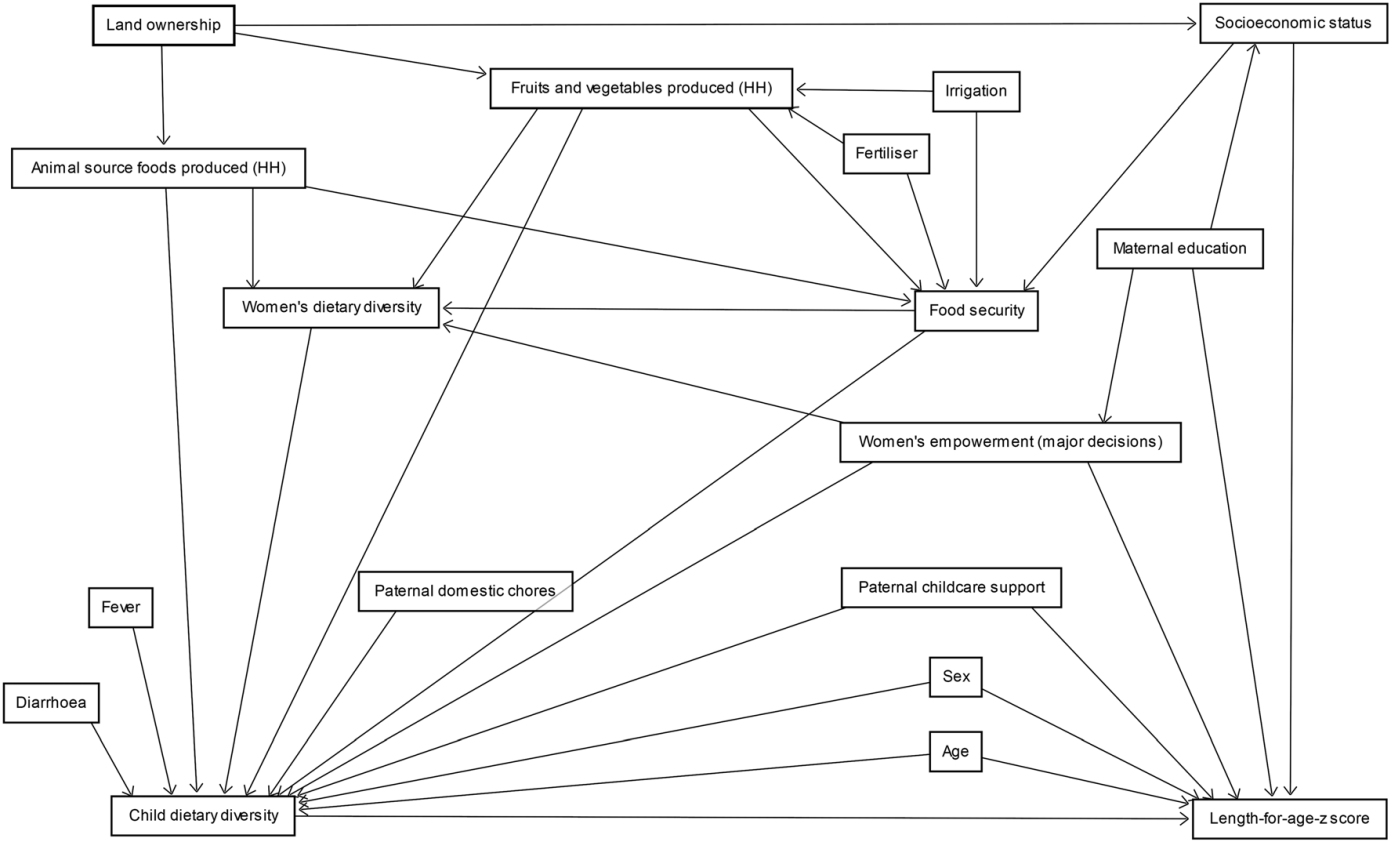
Directed acyclic graph (DAG) mapping causal relationships. DAGitty web‐based software was used to develop the diagram and to determine the minimal adjustment variable set to estimate total effect when regressing each explanatory risk factor on the outcome of interest—in this study, dietary diversity or length‐for‐age *z*‐score (Textor et al., 2016)

We hypothesised pathways of impact between risk factors and dietary diversity and linear growth in children. To represent these pathways, a DAG was developed using DAGitty software (Textor, van der Zander, Gilthorpe, Liśkiewicz, & Ellison, 2016; see Figure 1). DAGs represent a set of assumptions about the causal relationships between variables that are made by researchers based on evidence and logical reasoning and provide a basis on which to reduce bias in statistical modelling (Sauer & Vanderweele, 2013; Shrier & Platt, 2008). Recent evidence on agricultural production and women's empowerment factors supported construction of our diagram (Cunningham, Ruel, Ferguson, & Uauy, 2015; Hirvonen & Hoddinott, 2016; Ruel & Alderman, 2013). Occurrence of fever and diarrhoea in the past 2 weeks were hypothesised to influence child feeding practices but not growth due to the limited reference period.

Based on ancestor variables for each individual association, we used the DAGitty software to identify the unique set of covariates to be included in a multivariate model of that association. Unlike stepwise regression, covariates were not forced into a single multivariate model thus avoiding the table 2 fallacy (i.e., presentation of multiple adjusted effect estimates from a single model in a single table, often misunderstood to represent effects of the same direct causal type and therefore misinterpreted; Westreich & Greenland, 2013). We also decided a priori to add age and sex into all multivariate models, and age squared into multivariate model for LAZ. Associations between factors and child dietary diversity were investigated using univariate and multivariate ordinal logistic regression modelling. Associations between factors and LAZ were investigated using univariate and multivariate linear regression modelling. Robust standard errors were used to account for clustering at the *kebele* level. All analyses were conducted using Stata/IC (version 15).

### 3.6 Ethics

The baseline protocol was approved by the Scientific and Ethical Review Committee at EPHI (Ref number: SERO‐54‐3‐2016) and by the LSHTM ethics committee (Ref number: 10937) prior to data collection. By approval of both institutional review boards, informed written consent was given by caregivers of young children and witnessed by the local health extension worker.

## 4 RESULTS

A total of 1,848 children 6–23 months and their respective caregivers participated in the study. Of children 6–23 months of age, 6.0% consumed no complementary foods (breastfed only), 16.6% ate 1 food group, 31.1% ate 2 groups, 26.9% ate 3 groups, and 19.5% ate ≥4 groups. The number of food groups consumed increased with child age; the mean (*SD*) age of those children consuming no complementary foods was 11.1 (4.5) months and 15.7 (4.7) months among those consuming ≥4 food groups. Boys had lower LAZ (−1.23; *SD* 1.6) compared with girls (−1.05; *SD* 1.5), as did those of lower socio‐economic status (−1.29; *SD* 1.6) compared with those of higher status (−0.99; *SD* 1.5). Median maternal education was 3 years [IQR: 0, 7] for those children who consumed ≥4 food groups in comparison with 0 year of education [IQR: 0, 2] among those who consumed 0 food groups. Children with fathers who supported childcare daily or weekly consumed a higher number of food groups to those with less frequent paternal support. Complete socio‐economic and demographic characteristics of the participants are described in Table 1.

**Table 1:** Baseline socio‐economic and demographic characteristics of children 6–23 months (*n* = 1,848)

	Child dietary diversity, food groups					LAZ (*n* = 1,775)
Explanatory variable	0	1	2	3	4–7	Mean (*SD*)
*n* (%)	*n* = 106 (6.0)	*n* = 296 (16.6)	*n* = 555 (31.1)	*n* = 479 (26.9)	*n* = 347 (19.5)	
Children dietary diversity score, food groups (*n* = 1,783)						
0						−1.11 (1.5)
1						−1.18 (1.6)
2						−1.07 (1.6)
3						−1.23 (1.5)
4–7						−1.11 (1.5)
Age in months (mean, *SD*; *n* = 1,848)	11.1 (4.5)	12.4 (4.7)	14.5 (4.9)	15.5 (4.8)	15.6 (4.7)	−1.14 (1.5)
Sex (*n* = 1,847)						
Male	47 (44.3)	153 (51.7)	276 (49.7)	251 (52.4)	175 (50.4)	−1.23 (1.6)
Female	59 (55.7)	143 (48.3)	279 (50.3)	228 (47.6)	171 (49.3)	−1.05 (1.5)
SES (*n* = 1,848)						
Poorest	42 (39.6)	123 (41.6)	194 (35.0)	139 (29.0)	92 (26.5)	−1.29 (1.6)
Middle	36 (34.0)	96 (32.4)	197 (35.5)	176 (36.7)	91 (26.2)	−1.14 (1.5)
Richest	28 (26.4)	77 (26.0)	164 (29.6)	164 (34.2)	164 (47.3)	−0.99 (1.5)
Maternal education, years (median, IQR) (*n* = 1,848)	0 [0, 2]	0 [0, 4]	0 [0, 5]	0 [0, 4]	3 [0, 7]	−1.14 (1.5)
Decision making on major household purchases (*n* = 1,848)						
Father or others only	40 (37.7)	103 (34.8)	170 (30.6)	132 (27.6)	106 (30.6)	−1.27 (1.5)
Jointly or mother only	66 (62.3)	193 (65.2)	385 (69.4)	347 (69.4)	241 (69.5)	−1.08 (1.5)
Paternal childcare support (*n* = 1,752)						
Never	10 (9.8)	26 (9.4)	56 (10.6)	38 (8.3)	12 (3.7)	−1.16 (1.7)
Rarely	35 (34.3)	94 (33.9)	189 (35.6)	156 (34.2)	105 (32.2)	−1.17 (1.6)
Weekly or everyday	57 (55.9)	157 (56.7)	286 (53.9)	262 (57.5)	209 (64.1)	−1.12 (1.5)
Paternal domestic chores (*n* = 1,752)						
Never	43 (42.2)	109 (39.4)	227 (42.8)	190 (41.7)	123 (37.7)	−1.21 (1.6)
Rarely	25 (24.5)	82 (29.6)	133 (25.1)	107 (23.5)	89 (27.3)	−1.11 (1.5)
Weekly or everyday	34 (33.3)	86 (31.1)	171 (32.2)	159 (34.9)	114 (35.0)	−1.09 (1.5)
Food insecurity score (median, IQR) (*n* = 1,848)	0 [0, 7]	1 [0, 7]	0 [0, 6]	0 [0, 5]	0 [0, 3]	−1.14 (1.5)
Women's DD score (median, IQR) (*n* = 1,848)	3 [2, 4]	3 [2, 4]	3 [3, 4]	3 [3, 4]	4 [3, 4]	−1.14 (1.5)
Animal source food types produced, household (median, IQR) (*n* = 1,848)	1 [0, 3]	1 [0, 2]	1 [0, 2]	1 [0, 3]	1 [0, 3]	−1.14 (1.5)
Fruit and vegetable types produced, household (median, IQR) (*n* = 1,848)	1 [0, 2]	0 [0, 2]	1 [0, 2]	1 [0, 2]	1 [0, 2]	−1.14 (1.5)
Hectares (median, IQR) (*n* = 1,729)	0.5 [0, 1]	0.5 [0.25, 1]	0.5 [0.25, 1]	0.5 [0.25, 1]	0.5 [0.25, 1.5]	−1.14 (1.5)
Irrigation (*n* = 1,847)						
No	85 (80.2)	263 (88.9)	489 (88.1)	417 (87.2)	292 (84.2)	−1.14 (1.5)
Yes	21 (19.8)	33 (11.2)	66 (11.9)	61 (12.8)	55 (15.9)	−1.13 (1.6)
Fertiliser (*n* = 1,848)						
No	19 (17.9)	40 (13.5)	75 (13.5)	60 (12.5)	44 (12.7)	−1.09 (1.5)
Yes	87 (82.1)	256 (86.5)	480 (86.5)	419 (87.5)	303 (87.3)	−1.15 (1.5)
Diarrhoea, past 2 weeks (*n* = 1,783)						
No/do not know	68 (64.2)	192 (64.9)	422 (76.0)	339 (70.8)	246 (70.9)	−1.15 (1.5)
Yes	38 (35.9)	104 (35.1)	133 (24.0)	140 (29.2)	101 (29.1)	−1.11 (1.6)
Fever, past 2 weeks (*n* = 1,783)						
No/do not know	73 (68.9)	207 (69.9)	405 (73.0)	344 (71.8)	247 (71.2)	−1.12 (1.5)
Yes	33 (31.1)	89 (30.1)	150 (27.0)	135 (28.2)	100 (28.8)	−1.18 (1.5)

### 4.1 Dietary diversity

In adjusted models, children from the highest socio‐economic group had increased odds (adjusted *OR* 1.50; 95% CI [1.16, 1.93]; *P* = .004) of high dietary diversity compared with those in the lowest group (see Table 2). Other demographic factors positively associated with child dietary diversity included increase in age in months and land ownership. Cultivation of each additional type of fruit or vegetable within the household was associated with 16% higher odds of achieving high dietary diversity (adjusted *OR* 1.16 [1.09, 1.25], *P* < .001) and 6% higher odds for production of each animal source food (adjusted *OR* 1.06 [1.00, 1.13]; *P* = .06). Positive associations were also identified between dietary diversity and gender‐related variables including years of maternal education, women's empowerment (major decision‐making power), and paternal childcare support.

**Table 2:** Protective and risk factors for dietary diversity among children 6–23 months (*n* = 1,848)

Variable (*n,* multivariate models)	Unadjusted (*OR*)	CIs	*P* value	Adjusted (*OR*)	CIs	*P* value
Age (months) (*n* = 1,783)	1.11	[1.09, 1.13]	<.001	1.11^a^	[1.09, 1.13]	<.001
Sex (*n* = 1,782)						
Male	1		.07	1^b^		.05
Female	0.96	[0.81, 1.14]		0.94	[0.79, 1.12]	
Socio‐economic status (SES) (*n* = 1,667)						
Poorest	1		<.001	1^c^		.004
Middle	1.21	[0.98, 1.50]		1.07	[0.85, 1.35]	
Richest	1.86	[1.49, 2.33]		1.50	[1.16, 1.93]	
Maternal education (years) (*n* = 1,782)	1.07	[1.04, 1.09]	<.001	1.07^d^	[1.05, 1.10]	<.001
Women's decision‐making power—Major household expenses (*n* = 1,667)						
Husband/other	1		.05	1^e^		.02
Jointly/self	1.22	[1.00, 1.48]		1.27	[1.03, 1.55]	
Paternal childcare support (*n* = 1,691)						
Never	1		.02	1^d^		.02
Rarely	1.52	[1.08, 2.13]		1.52	[1.08, 2.14]	
Weekly/every day	1.61	[1.16, 2.22]		1.62	[1.17, 2.24]	
Paternal domestic chores (*n* = 1,691)						
Never	1		.59	1^d^		.64
Rarely	1.02	[0.82, 1.29]		1.03	[0.82, 1.30]	
Weekly/every day	1.11	[0.90, 1.38]		1.11	[0.89, 1.37]	
Food insecurity score (HFIAS) (*n* = 1,667)	0.96	[0.94, 0.98]	<.001	0.98^f^	(0.96, 1.00)	.04
Minimum women's dietary diversity (MDD‐W) (*n* = 1,782)	1.68	[1.53, 1.85]	<.001	1.63^g^	[1.48, 1.80]	<.001
Animal source food types produced, household (*n* = 1,667)	1.12	[1.05, 1.18]	<.001	1.06^h^	(1.00, 1.13)	.06
Fruit and vegetable types produced, household (*n* = 1,667)	1.16	[1.09, 1.23]	<.001	1.16^i^	[1.09, 1.24]	<.001
Land owned, hectares (*n* = 1,668)	1.11	[1.01, 1.21]	<.02	1.11^d^	[1.02, 1.21]	.02
Irrigation (*n* = 1,781)						
No	1		.44	1^d^		.50
Yes	1.12	[0.84, 1.48]		1.10	[0.83, 1.46]	
Fertiliser (*n* = 1,782)						
No	1		.22	1^d^		.17
Yes	1.18	[0.90, 1.55]		1.21	[0.92, 1.59]	
Diarrhoea, past 2 weeks (*n* = 1,782)						
No	1		.32			.33
Yes	0.91	[0.75, 1.10]		0.91^d^	[0.75, 1.10]	
Fever, past 2 weeks (*n* = 1,782)						
No	1		.79			.68
Yes	0.97	[0.80, 1.18]		0.96^d^	[0.79, 1.17]	

Variables adjusted for in multivariate ordinal logistic regression modelling:

a Sex.

b Age.

c Age, sex, maternal education, land ownership, women's empowerment, irrigation, fertiliser, fruit and vegetables produced (FV), and animal source foods produced (ASF).

d Age and sex.

e Age, sex, SES, maternal education, land ownership, food security, irrigation, fertiliser, FV, and ASF.

f Age, sex, SES, maternal education, land ownership, women's empowerment, irrigation, fertiliser, FV, and ASF.

g Age, sex, women's empowerment, food security, FV, and ASF.

h Age, sex, SES, maternal education, land ownership, women's empowerment, irrigation, fertiliser, and FV (*n* = 1,667).

i Age, sex, SES, maternal education, land ownership, women's empowerment, irrigation, fertiliser, and ASF.

### 4.2 Length‐for‐age

Child dietary diversity was positively associated with LAZ: children who consumed the highest number of food groups (between 4 and 7) had 0.42 higher mean LAZ (95% CI [0.08, 0.77]; *P* = .006) compared with those children who consumed no complementary foods (see Table 3). Girls had better growth compared with boys and other demographic predictors of LAZ included age, socio‐economic status, and land ownership. Household cultivation of fruit and vegetables was positively associated with LAZ (adjusted coefficient 0.05 [0.01, 0.10], *P* = .03). Weak evidence suggests that animal source foods may be negatively associated with growth. Maternal education was linked to higher LAZ, but no evidence of association was identified between LAZ and women's empowerment or father's support for childcare.

**Table 3:** Protective and risk factors for length‐for‐age *z*‐score among children 6–23 months (*n* = 1,848)

Variable (*n, multivariate models*)	Unadjusted coefficient	CIs	*P* value	Adjusted coefficient	CIs	*P* value
Child dietary diversity (food groups) (*n* = 1,579)						
0	1		.64	1^a^		.006
1	−0.11	[−0.45, 0.23]		0.08	[−0.26, 0.41]	
2	−0.02	[−0.33, 0.30]		0.42	[0.10, 0.75]	
3	−0.15	[−0.47, 0.17]		0.35	[0.02, 0.68]	
4–7	−0.05	[−0.38, 0.28]		0.42	[0.08, 0.77]	
Age (in months) (*n* = 1,775)	−0.25	[−0.34, −0.17]	<.001	−0.25^b^	[−0.34, −0.16]	<.001
Age squared (*n* = 1,775)	0.01	[0.003, 0.01]	<.001	0.005^b^	[0.002, 0.01]	<.001
Sex (*n* = 1,775)						
Male	1		.01	1^c^		.005
Female	0.18	[0.04, 0.32]		0.19	[0.06, 0.32]	
SES (*n* = 1,663)						
Poorest	1		.005	1^d^		.009
Middle	0.15	[−0.27, 0.32]		0.11	[−0.06, 0.29]	
Richest	0.30	[0.12, 0.48]		0.30	[0.10, 0.49]	
Maternal education, years (*n* = 1,775)	0.03	[0.01, 0.05]	.01	0.02^e^	[0.004, 0.04]	.02
Women's decision‐making power—Major household expenses (*n* = 1,783)						
Husband/other	1		.03	1		.13
Jointly/self	0.17	[0.01, 0.32]		0.11^f^	[−0.03, 0.26]	
Paternal domestic chores (*n* = 1,684)						
Never	1		.49	1^e^		.47
Rarely	0.08	[−0.10, 0.26]		0.08	[−0.09, 0.26]	
Weekly/every day	0.10	[−0.07, 0.27]		0.09	[−0.07, 0.26]	
Food insecurity score (HFIAS) (*n* = 1,663)	−0.02	[−0.03, −0.002]	.03	−0.01^g^	[−0.02, 0.01]	.38
Minimum dietary diversity‐women (MDD‐W) (*n* = 1,775)	0.04	[−0.03, 0.11]	.25	0.01^h^	[−0.06, 0.08]	.79
Animal source food types produced (*n* = 1,663)	−0.02	[−0.06, 0.03)	.50	−0.04^i^	[−0.09, 0.004]	.08
Fruit and vegetable types produced (*n* = 1,775)	0.06	[0.01, 0.11]	.01	0.05^j^	[0.005, 0.10]	.03
Land owned, hectares (*n* = 1,664)	0.07	[0.007, 0.14]	.03	0.08^e^	[0.02, 0.15]	.01

Variables adjusted for in multivariate linear regression modelling:

a Age, age squared, sex, SES, maternal education, women's empowerment, paternal childcare support, food security, land ownership, irrigation, fertiliser, fruit and vegetables produced (FV), and animal source foods produced (ASF).

b Sex.

c Age and age squared.

d Age, age squared, maternal education, land ownership, irrigation, fertiliser, FV, and ASF.

e Age, age squared, and sex.

f Age, age squared, sex, and maternal education.

g Age, age squared, sex, SES, maternal education, land ownership, irrigation, fertiliser, FV, and ASF (*n* = 1,663).

h Age, age squared, sex, women's empowerment, food security, FV, and ASF.

i Age, age squared, sex, SES, maternal education, land ownership, women's empowerment, irrigation, fertiliser, and FV.

j Age, age squared, sex, SES, maternal education, land ownership, women's empowerment, irrigation, fertiliser, and ASF.

## 5 DISCUSSION

In this study, we used novel methods—construction of DAGs to diagram causal relationships—to more precisely identify and estimate factors associated with child dietary diversity and LAZ among children 6–23 months old in rural agrarian regions of Ethiopia. Child dietary diversity was itself associated with increased LAZ. Socio‐economic status, maternal education, land ownership, and cultivation of fruits and vegetables were also positively associated with LAZ, whereas weak evidence suggested that animal source food production at household level may be a risk factor for decreased LAZ. Factors positively associated with child dietary diversity included age, socio‐economic status, land ownership, maternal education, women's empowerment, partner support for childcare, food security, household fruit and vegetable production, and women's dietary diversity.

The association between dietary diversity and linear growth in rural agrarian settings is well‐established (Amugsi, Dimbuene, Kimani‐Murage, Mberu, & Ezeh, 2017; Borkotoky, Unisa, & Gupta, 2018; Busert et al., 2016) and consumption of at least four of seven food groups (WHO/UNICEF) is an indicator of micronutrient adequacy (WHO et al., 2010). In this study, we found that growth was improved among children consuming two food groups or more compared with 0–1 food groups. The benefits from improved dietary diversity even at the very low levels may be partly explained by widespread challenges in Ethiopia with delayed initiation of complementary feeding, as observed in this study and in national statistics (51% of children 6–8 months do not yet consume complementary foods; Central Statistical Agency, 2016), and by high prevalence of religious fasting that prohibits adherents from eating animal source foods for more than 200 days per year (Desalegn, Lambert, Riedel, Negese, & Biesalski, 2018). Diversity of the diet also increased with child age in months, indicating that children of complementary feeding age were slow to begin consuming a wider range of food groups.

Access to a variety of foods is a prerequisite to good child dietary diversity. Our findings also confirm evidence from multiple studies showing that quality of children's diets worsens as food insecurity becomes more severe (Rodriguez, Mundo‐Rosas, Mendez‐Gomez‐Humaran, Perez‐Escamilla, & Shamah‐Levy, 2017). In the Ethiopian context, in which meals are largely shared, maternal dietary diversity may also partly serve as a proxy indicator of household access to diverse foods. A study conducted in Ghana showed that as the number of food groups consumed by mother increased, so too did the number consumed by their children (Amugsi, Mittelmark, & Oduro, 2015). Low socio‐economic status has been identified as a risk factor for low child dietary diversity in a wide range of contexts (Karwa, Godhia, & Jadhav, 2016; Rakotonirainy et al., 2018) and associations with stunted growth are well‐established (Adekanmbi, Kayode, & Uthman, 2013; Devakumar et al., 2018; Leroy, Habicht, Gonzalez de Cossio, & Ruel, 2014; Poda, Hsu, & Chao, 2017). Similarly, land ownership was also an important protective factor for child feeding practices in this study and others (Devakumar et al., 2018; Hailemariam, Girmay, & Girmay, 2018), perhaps due to the central role of agriculture in ensuring food security via food provision and/or income.

Agriculture‐related characteristics and practices may increase access to and use of nutrient‐rich food. We found strong evidence of an association between the cultivation of fruits and vegetables within the household and increases in both child dietary diversity and LAZ. Consumption of fruits and vegetables improves micronutrient status and immune function and may support child growth (Aguayo, Nair, Badgaiyan, & Krishna, 2016). A previous study in Ethiopia also reported that higher vegetable and fruit dietary consumption is associated with increased LAZ and reduced risk of stunting (Melaku et al., 2018), consistent with findings from Nepal and northern Ghana (Mulmi et al., 2017; Saaka, Osman, & Hoeschle‐Zeledon, 2017).

By contrast, we found weak suggestion of an inverse association between LAZ and animal source food production at household level. Poor environmental hygiene related to livestock husbandry has been documented in Ethiopia and in other developing contexts (Mosites et al., 2015). Keeping poultry in the household dwelling overnight, a common local practice, has been found to be associated with decreased child growth (Headey & Hirvonen, 2016). Despite some evidence from our study that production of animal source foods increases child dietary diversity, interventions that promote livestock rearing may need to include strong animal hygiene education and child feeding counselling to ensure that—in the Ethiopian social context—animal source foods are given with sufficient consistency and quantity to support child growth.

We investigated a range of gender‐related risk factors. Maternal education is a well‐established predictor of child feeding practices and nutritional status (Boyle et al., 2006; Wachs, Creed‐Kanashiro, Cueto, & Jacoby, 2005; Wang et al., 2017). A study conducted in Addis Ababa, Ethiopia, reported that mother's attainment of secondary education and above was associated with higher odds of achieving minimum dietary diversity among children 6–23 months old (Solomon, Aderaw, & Tegegne, 2017). Women's empowerment—measured in this study by women's power to decide major household expenses—was associated with child dietary diversity but not LAZ, consistent with other findings linking women's participation in financial decisions and quality of child diets (Amugsi, Lartey, Kimani‐Murage, & Mberu, 2016; Beyene, Worku, & Wassie, 2015). We also found that paternal childcare support predicted improved child diet quality. Mechanisms may include increased time for mothers to prepare food or increased prioritisation of children's needs when drawing on household resources. Ethiopian fathers' knowledge and support for child feeding practices has been shown to be positively associated with child dietary diversity (Bilal et al., 2016).

### 5.1 Strengths and limitations

In this study, we have extended investigation of factors beyond those commonly known or hypothesised to be associated with child diets and nutritional status to include some less‐researched factors: household agricultural diversity and gender‐related norms. Our analyses were based on a large dataset collected as primary data, and we used causal diagrams to represent our assumptions about the causal influences between the factors of interest and the outcomes. We then used these diagrams to identify variables to include in multivariable analysis. This approach allowed for improved estimates of the multiple primary associations between individual factors of interest and outcomes; we identified and adjusted for the unique set of covariates exerting direct or indirect effects on an individual association as per the causal relationships specified. This is an advance on “single model single table” multivariate modelling which displays—as a result of modelling a single primary association—figures representing a mixture of different types of causal effect estimates for covariates (secondary, direct and indirect) without explicit distinction or comparability between them (Westreich & Greenland, 2013).

Data were obtained from a single cross‐sectional study, and therefore, evidence from our analyses is limited by underlying assumptions of temporality. Dietary data may have been subject to recall bias and may not have shown usual intake as it was based on one 24‐hr recall. The data were also collected over a period inclusive both of the Lent fasting period before Easter and the meat‐heavy period directly following Easter. Other seasonal variation in the diet was not captured. Certain factors known be associated with stunting such as child birthweight and household sanitation were not included in this study.

### 5.2 Policy implications and further research

We showed that child diets in rural, agrarian regions of Ethiopia may be improved with increased household production of fruits and vegetables and of animal source foods. Interventions that seek to diversify farms such as agriculture extension advising, homestead gardening promotion, and/or provision of seed or animal inputs may be effective (Hirvonen & Headey, 2018; Sibhatu, Krishna, & Qaim, 2015). Other means of increasing access to and use of diverse foods might include market‐based initiatives or voucher or cash interventions combined with nutrition education and promotion (Ruel & Alderman, 2013). Interventions to increase livestock rearing at household level may help to improve dietary diversity. Further research is needed on the complex relationships between livestock rearing, hygiene practices, consumption of animal source foods, and child growth (Mosites et al., 2015).

Child feeding was also linked to paternal childcare support and women's empowerment, suggesting that inclusion of husbands and other community members in nutrition education initiatives—as part of a broader social and behaviour change communication strategy—may be effective to improve child diets (Nguyen et al., 2018). However, strategies to effectively engage men and community influencers are not yet well researched (Lutter et al., 2013). Education of girls remains essential to sustained improvement in child growth in Ethiopia (Lailulo, Sathiya Susuman, & Blignaut, 2015).

## 6 CONCLUSIONS

Young children in Ethiopia are slow to begin consuming a range of diverse complementary foods. Household cultivation of fruits and vegetables in rural agrarian settings can improve both child feeding practice and linear growth. Gender‐sensitive interventions to increase maternal education, women's empowerment, and paternal support for childcare may support improved diversity of child feeding.

## CONFLICTS OF INTEREST

The authors declare that they have no conflict of interest.

## CONTRIBUTIONS

DK, CM, MS, AD, and EA conceived the idea and designed the study. DK, SE, GA, MS, and CM coordinated and supervised training and data collection. DK, GA, SE, CM, MS, and EA analysed the data and interpreted. DK and CM wrote the first draft of the manuscript. AD, CM, MS, and EA critically reviewed the manuscript. EA provided technical support in analysis. All authors reviewed and approved the manuscript.

## Supporting information


**Data S1.** Sustainable Undernutrition Reduction in Ethiopia (SURE) Household Survey Baseline Questionnaire V 1.11
