# Supplementary material for: Factors associated with dietary diversity and length‐for‐age z‐score in rural Ethiopian children aged 6–23 months: A novel approach to the analysis of baseline data from the Sustainable Undernutrition Reduction in Ethiopia evaluation
Source: Matern Child Nutr. 2019 Jul 13;16(1):e12852. doi: 10.1111/mcn.12852 (PMC7038872; doi:10.1111/mcn.12852)
Supplement: Supplementary file 1 — Data S1. Sustainable Undernutrition Reduction in Ethiopia (SURE) Household Survey Baseline Questionnaire V 1.11 [file MCN-16-e12852-s001.pdf]

SUSTAINABLE UNDERNUTRITION REDUCTION IN ETHIOPIA (SURE)

HOUSEHOLD SURVEY BASELINE QUESTIONNAIRE V 1.11

Module 1 – Household Identifiers and Listings

100	Region Code	|___|___|
101	Zone Code	|___|___|
102	Woreda code	|___|___|
103	Kebele code	|___|___|___|
104	Gote code	|___|___|___|
105	Household number<br>***Enter the household number***	|___|___|___|
106	Unique household ID<br>***To be copied onto all documents, e.g. consent forms***	|___|___|| / |___|___|___| / |___|___|___| / |___|___|___| Woreda / Kebele / Gote / Household

Interviewer's code	|___|___|___|	|___|___|___|	|___|___|___|
Result (Enter relevant code below)<br>1. Accepted<br>2. Refused	|__|	|__|	|__|

107		**Mother(s) of eligible children in the household**<br>Identify the mother or caregiver of each selected child 0-47 months. Read the consent form and answer any questions. Ask each mother/caregiver if she consents to be interviewed. If yes, complete the consent form and continue the first interview. If consent is not given, end the interview and continue to the next interview or household.				
**Person number**<br><br>**A**	**Name of mother/caregiver**<br><br>**B**		**Age in years**<br><br>**C**	**Years of formal education completed**<br>**D**	**Marital status**<br>**1=Single**<br>**2=Married**<br>**3=Divorced**<br>**4=Separated**<br>**5=Widowed**<br><br>**E**	**Consent given?**<br>**1=yes**<br>**0= no**<br><br>**F**
**10**	|___|___|___|___|___|___|___|___|___|___|___|		|___|___|	|__|__|	|__|	|__|
**11**	|___|___|___|___|___|___|___|___|___|___|___|		|___|___|	|__|__|	|__|	|__|
**12**	|___|___|___|___|___|___|___|___|___|___|___|		|___|___|	|__|__|	|__|	|__|
**13**	|___|___|___|___|___|___|___|___|___|___|___|		|___|___|	|__|__|	|__|	|__|
**14**	|___|___|___|___|___|___|___|___|___|___|___|		|___|___|	|__|__|	|__|	|__|

108	**Household Child Listing**
	Now I would like to ask you about all the children under 5 years of age who are residents of this house, to which you are the mother/primary caregiver. By resident in the house, I mean children who have been present for the last 3 months and who normally eat together. **Start with the oldest child (aged 5 years or younger).** Can you tell me the names of each of these children? *Record names in column B.*<br><br>➔ Do you have health/vaccination cards with the children's birthdates recorded?<br>*If the health/vaccination card is shown and the respondent confirms the information is correct (years and months),* ***record the dates of birth*** *below in column D. If the respondent does not confirm the information on the card,* **resolve inconsistences***.*<br><br>*If there is no health/vaccination card, ask the respondent the* ***exact date of birth****, if known, and record in column D. Then calculate the child's age in years and months and record in columns E and F, respectively. If the exact date of birth is unknown, then* **ask the age of the child in years and in months***. Record in columns E and F.*<br><br>*Child selection (****Column K****): If there is no more than one child per age category (0-5 months, 6-23 months, 24-47 months), select each child in column K. If there is more than one child in any given age group, randomly select one child for the group.*

Child Number A	Child Name<br>B	Sex<br>C	Date of Birth<br>D	Age in years<br>E	For each child 0-47m, enter age in months<br>F	Eligible child 0-5m?<br>G	Eligible child 6-23 m?<br>H	Eligible child 24-47m?<br>I	Mother or caregiver of child<br>J	Child selected<br>K
	**Enter First Name (If Identical names in the same household use the initial of the last name)**	**1 = M<br>2 = F**	**(dd/mm/yyyy)**	**If less than one year, enter 0**	**Record age in months**	**0 = No<br>1 = Yes**	**0 = No<br>1 = Yes**	**0 = No<br>1 = Yes**	**Record Person Number from 107 (col A)**	**0 = No<br>1= Yes**
1	|__|__|__|__|__|__|__|__|__|__|__|__|	|__|	|__|__| / |__|__| / |__|__|__|__|	|__|	|__|__|	|__|	|__|	|__|	|__|__|	|__|
2	|__|__|__|__|__|__|__|__|__|__|__|__|	|__|	|__|__| / |__|__| / |__|__|__|__|	|__|	|__|__|	|__|	|__|	|__|	|__|__|	|__|
3	|__|__|__|__|__|__|__|__|__|__|__|__|	|__|	|__|__| / |__|__| / |__|__|__|__|	|__|	|__|__|	|__|	|__|	|__|	|__|__|	|__|
4	|__|__|__|__|__|__|__|__|__|__|__|__|	|__|	|__|__| / |__|__| / |__|__|__|__|	|__|	|__|__|	|__|	|__|	|__|	|__|__|	|__|
5	|__|__|__|__|__|__|__|__|__|__|__|__|	|__|	|__|__| / |__|__| / |__|__|__|__|	|__|	|__|__|	|__|	|__|	|__|	|__|__|	|__|
6	|__|__|__|__|__|__|__|__|__|__|__|__|	|__|	|__|__| / |__|__| / |__|__|__|__|	|__|	|__|__|	|__|	|__|	|__|	|__|__|	|__|
7	|__|__|__|__|__|__|__|__|__|__|__|__|	|__|	|__|__| / |__|__| / |__|__|__|__|	|__|	|__|__|	|__|	|__|	|__|	|__|__|	|__|
	**TOTAL**					|__|	|__|	|__|		

Module 2: Individual Child Information

FOR CHILDREN 0-47 MONTHS

CHILD INFORMATION
(this information is entered after identifying eligible children from the household child listing, 108)

Separate section should be completed for each eligible child aged 0-47 months.

Name of Child (from column B of 108): |___|___|___|___|___|___|___|___|___|___|___|

Individual Child Code: |___|___|___| – |___|___| – |___|
Household number (105) – Mother Number (col A 107) – Child Number (col A 108)

Name of mother/caregiver (from column B of 107): |___|___|___|___|___|___|___|___|___|___|___|___|___|___|

What is the age of this child in months? (column F of 108): |___|___|
If the age of child (NAME) > 23 months → Skip to 248

Module 2 Section 1 – Infant and young child feeding

FOR CHILDREN 0-23 MONTHS

This module is to be administered to the mother/caregiver of children ages 0-23 months. Verify that the respondent you are speaking to is the mother/caregiver of the child listed in the above Child Information

200	***For the interviewer:*** ***Is the respondent verified as the mother or caregiver of this child (NAME)?*** ***If the mother/caregiver is not available, you must end the interview and make an appointment to visit the house again.***	**1 = Yes**<br>**0 = No → End the interview and return later**	|__|
201	Has ***(NAME)*** ever been breastfed?	**1 = Yes**<br>**0 = No → GO to 205**<br>**98 = Don't know → GO to 205**	|___|___|
202	How long after birth did you first put ***(NAME)*** to the breast, even if your breast milk did not arrive?	**1 = Immediately after birth, or within 1 hour**<br>**2 = Between 1 and 24 hours**<br>**3 = More than 24 hours after delivery**<br>**98 = Don't know**	|___|___|
203	Was ***(NAME)*** breastfed yesterday from sunrise until today sunrise?	**1 = Yes**<br>**0 = No**<br>**98 = Don't know**	|___|___|
204	Sometimes babies are fed breast milk in different ways, for example by spoon, cup or bottle. This can	**1 = Yes**<br>**0 = No**	|___|___|

	happen when the mother cannot always be with her baby. Sometimes babies are breastfed by another woman, or given breast milk from another woman by spoon, cup or bottle or some other way. This can happen if a mother cannot breastfeed her own baby.<br><br>Did ***(NAME)*** consume breast milk in any of these ways yesterday from sunrise until today sunrise?		**98 = Don't know**	
205	Now I would like to ask you about some medicines and vitamins that are sometimes given to infants.<br><br>Was ***(NAME)*** given any vitamin drops or other medicines as drops yesterday from sunrise until today sunrise?		**1 = Yes**<br>**0 = No**<br>**98 = Don't know**	|___|___|
206	Was ***(NAME)*** given [*Local name for ORS*] yesterday from sunrise until today sunrise?<br><br>**<u>SHOW ORS PACKET.</u>**		**1 = Yes**<br>**0 = No**<br>**98 = Don't know**	|___|___|
	Next I would like to ask you about some liquids that ***(NAME)*** may have had yesterday from sunrise until today sunrise?<br><br>**Read list of Liquids starting with 'plain water'.**		Did ***(NAME)*** have any ***(item from list)***?:<br>**1 = yes**<br>**0 = No**<br>**98 = Don't know**	How many times yesterday during the day or at night did ***(NAME)*** consume any ***(item from list)***?<br>*(Record number)*<br>**98 = Don't know**
207	**Liquids**	Plain water?	|___|___|	
208		Infant formula such as [**Nestle, Anchor…**]?	|___|___|	|___|___|
209		Milk such as tinned, powdered, or fresh animal milk?	|___|___|	|___|___|
210		Juice or juice drinks?	|___|___|	
211		Clear broth?	|___|___|	
212		Yogurt?	|___|___|	|___|___|
213		Thin porridge?	|___|___|	
214		Any other liquids such as [**list other water- based liquids available in the local setting**]?	|___|___|	
215		Any other liquids (except ORS, medicines, vitamins)?	|___|___|	

	Please describe everything that ***(NAME)*** ate yesterday since sunrise to today sunrise, whether at home or outside the home.<br><br>**Ask:** When ***(NAME)*** woke up yesterday, did ***(NAME)*** eat anything at that time?<br>- **If yes:** Please tell me everything ***(NAME)*** ate at that time<br>- **Probe***:* anything else? **Until respondent says nothing else. if no, continue to next question**<br><br>**Ask:** What did ***(NAME)*** do after that? Did ***(NAME)*** eat anything at that time?<br>- **If yes***:* Please tell me everything ***(NAME)*** ate at that time.<br>- **Probe:** anything else? **Until respondent says nothing else.**

	**Continue asking what *(NAME)* did/ate, until respondent says the child went to sleep until the next day sunrise.**<br><br>**If respondent mentions mixed dishes like porridge, sauce or stew.**<br>- **Ask:** What ingredients were in that **(mixed dish)**?<br>- **Probe***:* anything else? **Until respondent says nothing else.**<br><br>➔ **If foods are used in small amounts for seasoning or as a condiment, include them under the condiments food group.**
216	**OTHER FOODS: Please write down other foods in this box that respondent mentioned but are not in the list below**

**Food groups**		**Eaten?**<br>**1 = Yes**<br>**0 = No**
217	Porridge, bread, rice, noodles, or other foods made from grains	**|__|**
218	Pumpkin, carrots, squash, or sweet potatoes that are yellow or orange inside	**|__|**
219	White potatoes, white yams, cassava, or any other foods made from roots	**|__|**
220	Any dark green leafy vegetables (kale, dark green lettuce, moringa …)	**|__|**
221	Ripe mangoes, ripe papayas(**insert other local vitamin a-rich fruits**)	**|__|**
222	Any other fruits or vegetables	**|__|**
223	Liver, kidney, heart, or other organ meats	**|__|**
224	Any meat, such as beef, pork, lamb, goat, chicken	**|__|**
225	Eggs	**|__|**
226	Fresh or dried fish, shellfish, or seafood	**|__|**
227	Any foods made from beans, peas, lentils, nuts, or seeds	**|__|**
228	Cheese, yogurt, or other milk products	**|__|**
229	Any oil, fats, or butter, or foods made with any of these	**|__|**
230	Any sugary foods such as chocolates, sweets, candies, pastries, cakes, or biscuits	**|__|**
231	Condiments for flavour, such as chillies, spices, herbs,	**|__|**

Once the respondent finishes recalling foods eaten, read each food group which were coded as 'no', ask: Yesterday during the day or night, did ***(NAME)*** drink/eat any (***food group items)?*** **Fill answer in corresponding food item*.***

Check categories 217-231. If all no, → go to 232
If at least one yes, → go to 233

232	Did ***(NAME)*** eat any solid, semi-solid, or soft foods yesterday from sunrise until today sunrise?<br><br>**If 'yes' probe***:* What kind of solid, semi-solid, or soft foods did ***(NAME)*** eat?	**1 = Yes -> fill in table above, then continue with 233**<br>**0 = No → GO to 235**<br>**98 = Don't know → GO to 235**	|___|___|
233	How many times did ***(NAME)*** eat solid, semi- solid, or soft foods other than liquids yesterday from sunrise until today sunrise?	**Fill in number of times.**<br>**98 = Don't know**	|___|___|
234	From sunrise yesterday to today sunrise, was ***(NAME)*** eating animal source foods (fasting)?	**1 = Yes**<br>**0 = No**	|___|___|
235	Did ***(NAME)*** drink anything from a bottle with a nipple yesterday during the day or night?	**1 = Yes**<br>**0 = No**<br>**98 = Don't know**	|___|___|

236	To your knowledge, for how long does a child need to be given **ONLY** breast-milk/mother's milk and nothing else **(Not even water)**?<br><br>DO NOT PROMPT	**1 = First 3 months or less**<br>**2 = 3-5 months**<br>**3 = Up to 6 months**<br>**4 = From 6 months up to 1 year**<br>**5 = 13-23 months**<br>**6 = Never**<br>**98 = Don't know**<br>**99 = Other**	|___|___|
237	To your knowledge, at what age should a child **begin** eating soft, semi-solid or solid foods and other liquids, in addition to breastmilk?<br><br>DO NOT PROMPT	**1 = First 3 months or less**<br>**2 = 3-5 months**<br>**3 = At 6 months**<br>**4 = More 6 months up to 1 year**<br>**5 = 13-23 months**<br>**98 = Don't know**<br>**99 = Other**	|___|___|

**I am going to read you some statements about breastfeeding and complementary feeding made by other mothers who live in a community like yours. Please tell me if you agree with these statements.**<br><br>**Remember, there are no correct answers! I would like to know your opinion.**				
Breastfeeding	238	A child who is four months old does not need to eat anything or drink anything, not even water, other than breast milk	**1 = Yes**<br>**0 = No**<br>**98 = Don't know**	|___|___|
	239	If a child is sick (for example has fever/diarrhoea) breastfeeding must be stopped.	**1 = Yes**<br>**0 = No**<br>**98 = Don't know**	|___|___|
Complementary feeding	240	It is good for a child older than 6 months to eat eggs, cow milk, or meat even on fasting days	**1 = Yes**<br>**0 = No**<br>**98 = Don't know**	|___|___|
	241	It is dangerous to give meat to a child 6 months to a year old	**1 = Yes**<br>**0 = No**<br>**98 = Don't know**	|___|___|
	242	Food for a child over 1 year should be prepared separately from the other family members	**1 = Yes**<br>**0 = No**<br>**98 = Don't know**	|___|___|
	243	Eating a variety of foods is not necessary until children are old enough to go to school	**1 = Yes**<br>**0 = No**<br>**98 = Don't know**	|___|___|

Module 2 Section 2 – Services

FOR CHILDREN 6-47 MONTHS			
244	Within the last six months has ***(NAME)*** received a vitamin A dose like this? **SHOW CAPSULES.**	**1 = Yes**<br>**0 = No**<br>**98 = Don't know**	|___|___|
245	Has ***(NAME)*** had diarrhoea in the last 2 weeks?	**1 = Yes**<br>**0 = No → GO to 247**<br>**98 = Don't know → GO to 247**	|___|___|
246	Did you seek advice or treatment for the diarrhoea at a health post or from a HEW?<br><br>Note: If the problem is still exists, refer the child to nearby health post.	**1 = Yes**<br>**0 = No**<br>**98 = Don't know**	|___|___|
247	Has ***(NAME)*** been ill with a fever at any time in the last 2 weeks	**1 = Yes**<br>**0 = No → GO to 249**<br>**98 = Don't know → GO to 249**	|___|___|
248	Did you seek advice or treatment for the fever at a health post or from a HEW?<br><br>Note: If the problem is still exists, refer the child to nearby health post.	**1 = Yes**<br>**0 = No**<br>**98 = Don't know**	|___|___|
249	Has ***(NAME)*** had an illness with a cough at any time in the last 2 weeks?	**1 = Yes**<br>**0 = No → GO to 251**<br>**98 = Don't know → GO to 251**	|___|___|
250	Did you seek advice or treatment at a health post or from a HEW?<br><br>Note: If the problem is still exists, refer the child to nearby health post.	**1 = Yes**<br>**0 = No**<br>**98 = Don't know**	|___|___|

FOR CHILDREN 6-23 MONTHS			
251	Has your child been weighed at Growth Monitoring and Promotion <u>in the community</u> in the past 30 days? (everyone brought their child)	**1 = Yes**<br>**0 = No → GO to 253**<br>**98 = Don't know → GO to 253**	|___|___|
252	When you child was weighed at GMP, did you receive messages from HEW about how to feed your child?	**1 = Yes**<br>**0 = No**<br>**98 = Don't know**	|___|___|
253	Have you attended a women's only dialogue group in the past 60 days to discuss child feeding and/or agriculture?	**1 = Yes**<br>**0 = No**<br>**98 = Don't know**	|___|___|
254	Has your child attended MUAC screening in the community in the past 6 months?<br>***SHOW MUAC TAPE***	**1 = Yes**<br>**0 = No**<br>**98 = Don't know**	|___|___|
255	In the past 3 months, have you been visited at your household by an HEW and AEW <u>jointly</u>?	**1 = Yes**<br>**0 = No → GO to 300**<br>**98 = Don't know → GO to 300**	|___|___|

During the joint HEW/AEW visits, what child feeding topics were	256	My child's age	**For each mentioned:**<br><br>**1 = Yes**<br>**0 = No**	**|__|**
	257	Whether my child has been recently sick		**|__|**
	258	Exclusive breastfeeding for child <6 months		**|__|**
	259	Continued breastfeeding for child >6 months		**|__|**
	260	Beginning complementary feeding at 6 months		**|__|**
	261	How to make a thick porridge for child >6 months		**|__|**

discussed with you?<br><br>DO NOT PROMPT	262	Adding an egg, milk, dried meat, vegetables or other special foods to child's porridge		|__|
	263	Feeding diverse foods		|__|
	264	Meal frequency		|__|
	265	Feeding snacks		|__|
	266	Feeding children over 1 year from the family's food		|__|
	267	Hand washing		|__|
268	During the visit, were you referred to other health services (such as GMP, immunisations, vitamin A, wellness visits)?		**1 = Yes**<br>**0 = No**<br>**98 = Don't know**	|___|___|
During the joint HEW/AEW visits, what agriculture topics were discussed with you?<br><br>DO NOT PROMPT	269	Producing and eating diverse foods	**For each mentioned:**<br><br>**1 = Yes**<br>**0 = No**	|__|
	270	Crop selection		|__|
	271	Livestock		|__|
	272	Intercropping		|__|
	273	Methods to reduce soil erosion		|__|
	274	Using manure or other fertiliser		|__|
	275	Irrigation or water harvesting		|__|
	276	Protecting house and water sources from livestock and poultry		|__|
	277	Consuming eggs, milk and animal products		|__|
	278	Using income to buy nutritious foods or for medical care		|__|
	279	Women's participation in agriculture		|__|
	280	Role of men and women to support family's diet		|__|
281	Were your referred to other agricultural services during your household visit (such as famer's demonstration centres, farmer's cooperatives)?		**1 = Yes**<br>**0 = No**<br>**98 = Don't know**	|___|___|

Module 3 – Women's Empowerment

ABOUT MOTHERS

300	***Identify the most senior mother of the mothers who have a selected child. She is the mother who should respond to the rest of this interview from this point.***	**Person number (from Mother's Listing 108A)**	|__||__|
301	In the past major growing season (Meher) and minor growing (Belg) season, not including the current season, did you work on the family farm?	**1 = Yes**<br>**0 = No → Go to 309**	|__|

What sort of work did you do on the family farm?<br><br>READ THE LIST.	302	1 = Home (kitchen) gardening	**1 = Yes**<br>**0 = No**	|__|
	303	2 = Field work		|__|
	304	3 = Cash crop farming		|__|
	305	4 = Producing eggs or dairy		|__|
	306	5 = Raising livestock		|__|
	307	5 = Fishpond/ aquaculture		|__|
	308	7 = Other …………………..		|__|

309	From the work that you did on the farm did your household earn any money?	**1 = Yes**<br>**0 = No → Skip to 311**<br>**98 = Don't know**	|___|___|
310	Who usually decides how the money you earn will be used?<br><br>READ THE LIST.	**1 = Self**<br>**2 = Husband**<br>**3 = Self and husband jointly**<br>**4 = Someone else**	|__|
311	Who usually decides how the money your husband earns will be used?<br><br>READ THE LIST.	**1 = Self**<br>**2 = Husband**<br>**3 = Self and husband jointly**<br>**4 = Someone else**	|__|
312	Who usually makes decisions about major household purchases/sell such as cattle or livestock?<br><br>READ THE LIST.	**1 = Self**<br>**2 = Husband**<br>**3 = Self and husband jointly**<br>**4 = Someone else**	|__|
313	Who usually makes decisions about minor household purchases/sell such as spices/oils, soap, utensils, or daily household needs?<br><br>READ THE LIST.	**1 = Self**<br>**2 = Husband**<br>**3 = Self and husband jointly**<br>**4 = Someone else**	|__|
314	Who usually makes decisions about health care for your children?<br><br>READ THE LIST.	**1 = Self**<br>**2 = Husband**<br>**3 = Self and husband jointly**<br>**4 = Someone else**	|__|
315	Does your husband help you care for the children?	**1 = Yes**<br>**0 = No → Skip to 317**	|__|

316	Does he help care for the children almost every day, at least once a week, or rarely?	**1 = Every day**<br>**2 = At least once a week**<br>**3 = Rarely**	|__|
317	Does your husband help you with household chores like cooking, cleaning the house, fetching water, collecting firewood or other domestic work?	**1 = Yes**<br>**0 = No → Skip to 400**	|__|
318	Does he help almost every day, at least once a week, or rarely?	**1 = Every day**<br>**2 = At least once a week**<br>**3 = Rarely**	|__|

Module 4 – Household Food security and dietary diversity

ABOUT THE HOUSEHOLDS			
401	In the past four weeks, did you worry that your household would not have enough food?	**1 = Yes**<br>**0 = No → Go to 403**	|__|
402	How often did this happen?	**1= Rarely (once or twice in the past four weeks)**<br>**2 = Sometimes (three to ten times in the past four weeks)**<br>**3 = Often (more than ten times in the past four weeks)**	|__|
403	In the past four weeks, were you or any household member not able to eat the kinds of foods you preferred because of a lack of resources?	**1 = Yes**<br>**0 = No → Go to 405**	|__|
404	How often did this happen?	**1= Rarely (once or twice in the past four weeks)**<br>**2 = Sometimes (three to ten times in the past four weeks)**<br>**3 = Often (more than ten times in the past four weeks)**	|__|
405	In the past four weeks, did you or any household member have to eat a limited variety of foods due to a lack of resources?	**1 = Yes**<br>**0 = No → Go to 407**	|__|
406	How often did this happen?	**1= Rarely (once or twice in the past four weeks)**<br>**2 = Sometimes (three to ten times in the past four weeks)**<br>**3 = Often (more than ten times in the past four weeks)**	|__|
407	In the past four weeks, did you or any household member have to eat some foods that you really did not want to eat because of a lack of resources to obtain other types of food?	**1 = Yes**<br>**0 = No → Go to 409**	|__|
408	How often did this happen?	**1= Rarely (once or twice in the past four weeks)**<br>**2 = Sometimes (three to ten times in the past four weeks)**<br>**3 = Often (more than ten times in the past four weeks)**	|__|
409	In the past four weeks, did you or any household member have to eat a smaller meal than you felt you needed because there was not enough food?	**1 = Yes**<br>**0 = No → Go to 411**	|__|

410	How often did this happen?	**1= Rarely (once or twice in the past four weeks)<br>2 = Sometimes (three to ten times in the past four weeks)<br>3 = Often (more than ten times in the past four weeks)**	**|__|**
411	In the past four weeks, did you or any other household member have to eat fewer meals in a day because there was not enough food?	**1 = Yes<br>0 = No → Go to 413**	**|__|**
412	How often did this happen?	**1= Rarely (once or twice in the past four weeks)<br>2 = Sometimes (three to ten times in the past four weeks)<br>3 = Often (more than ten times in the past four weeks)**	**|__|**
413	In the past four weeks, was there ever no food to eat of any kind in your household because of lack of resources to get food?	**1 = Yes<br>0 = No → Go to 415**	**|__|**
414	How often did this happen?	**1= Rarely (once or twice in the past four weeks)<br>2 = Sometimes (three to ten times in the past four weeks)<br>3 = Often (more than ten times in the past four weeks)**	**|__|**
415	In the past four weeks, did you or any household member go to sleep at night hungry because there was not enough food?	**1 = Yes<br>0 = No → Go to 417**	**|__|**
416	How often did this happen?	**1= Rarely (once or twice in the past four weeks)<br>2 = Sometimes (three to ten times in the past four weeks)<br>3 = Often (more than ten times in the past four weeks)**	**|__|**
417	In the past four weeks, did you or any household member go a whole day and night without eating anything because there was not enough food?	**1 = Yes<br>0 = No → Go to 419**	**|__|**
418	How often did this happen?	**1= Rarely (once or twice in the past four weeks)<br>2 = Sometimes (three to ten times in the past four weeks)<br>3 = Often (more than ten times in the past four weeks)**	**|__|**

ABOUT THE MOTHER

Please describe the foods (meals and snacks) that you (the mother respondent) ate yesterday from sunrise to today sunrise, including foods purchased eaten outside of the home. Start with the first food eaten in the morning.

When the respondent recall is complete, fill in the food groups based on the information recorded above. For any food groups not mentioned, ask the respondent if a food item from this group was consumed.

➔ **Write down all food and drinks mentioned by the respondent. When the respondent has finished, probe for meals and snacks not mentioned.**

Breakfast	Snack	Lunch	Snack	Dinner	Snack

Question number	Food group	Examples	**1 = Yes**<br>**0 = No**
419	CEREALS	corn/maize, teff, rice, wheat, sorghum, millet or any other grains or foods made from these (e.g. bread, noodles, porridge or other grain products) e.g. [illegible]	|__|
420	VITAMIN A RICH VEGETABLES AND TUBERS	pumpkin, carrots, squash, or sweet potatoes that are orange inside + *other locally available vitamin-A rich vegetables (e.g. red pepper)*	|__|
421	WHITE TUBERS AND ROOTS	white potatoes, false banana (enset), white yams, white cassava, or other foods made from roots	|__|
422	DARK GREEN LEAFY VEGETABLES	dark green/leafy vegetables, including wild ones + *locally available vitamin-A rich leaves such as amaranth, , kale, spinach, pumpkin leaves, etc.*	|__|
423	OTHER VEGETABLES	other vegetables (e.g. tomato, onion, eggplant) , including wild vegetables	|__|
424	VITAMIN A RICH FRUITS	ripe mangoes, cantaloupe, apricots (fresh or dried), ripe papaya, dried peaches + *other locally available vitamin A-rich fruits*	|__|
425	OTHER FRUITS	other fruits, including wild fruits	|__|
426	ORGAN MEAT (IRON-RICH)	liver, kidney, heart or other organ meats or blood-based foods	|__|
427	FLESH MEATS	beef, pork, lamb, goat, chicken, or other birds	|__|
428	EGGS	chicken, duck, guinea hen or any other egg	|__|
429	FISH	fresh or dried fish	|__|
430	LEGUMES	beans, peas, lentils, chickpea, or foods made from these	|__|
430-a	NUTS AND SEEDS	nuts, seeds	|__|
431	MILK AND MILK PRODUCTS	milk, cheese, yogurt or other milk products	|__|
432	OILS AND FATS	oil, fats or butter added to food or used for cooking	|__|
433	SWEETS	sugar, honey, sweetened soda or sugary foods such as chocolates, candies, cookies and cakes	|__|

434	SPICES, CONDIMENTS, BEVERAGES	Spices (black pepper, salt), condiments (soy sauce, hot sauce), coffee, tea, alcoholic beverages OR *local examples: tela, tej, bordea, arkea, cheka, tselo, keneto…*	|__|
435	Did you eat anything (meal or snack) OUTSIDE of the home yesterday?		|__|
436	Did you fast yesterday during the day or night?		|__|
437	At any time during your most recent pregnancy, did you take iron folate supplements?<br><br>**SHOW IRON FOLATE TABLET**.		|__|

438	Do you have knowledge or awareness of food groups?	**0=No → Skip to 440**<br>**1=Yes**	|__|
439	To your knowledge, foods from how many different food groups should be eaten during a single meal by the family?<br><br>***<u>SHOW FOOD GROUP POSTER.</u>***	**1 = One**<br>**2 = Two**<br>**3 = Three**<br>**4 = Four or more**<br>**98 = Don't know**	|__||__|

To your knowledge, what are the benefits of eating many different types of food each day?<br><br>DO NOT PROMPT.	**For each mentioned: 1=Yes 0=No**		
	440	For good health / Prevent illness	|__||__|
	441	To help children grow well	|__||__|
	442	Other	|__||__|
	443	Don't know	|__||__|

Module 5 – Agriculture practices

**ABOUT THE HOUSEHOLD**			
500	Does any member of the household own any agricultural land?	**1 = Yes**<br>**0 = No → GO to 502**	|__|
501	How many hectares of agricultural land do members of this household own?<br><br>*Note: Convert local land measurement unit into hector after discussing with agriculture focal person/AEW.*	**Enter total number of hectares (If less than 1, Enter in decimals (example 0.5)**<br>**Enter 9999 if hectares are not known**	|__||__|.|__||__|

In the past 2 growing seasons (Meher and Belg), not including the current season, please describe all the crops (cereals, legumes, vegetables, fruits, seeds, and other crops) grown on your household farm.

→Then ask about how much was sold, consumed or used for another purpose.

Group		Crop	Did HH cultivate crop?<br>1 = yes<br>0 = No<br><br>*(If no, skip to the next item)*<br><br>A	During the previous Major seasons (Meher) and Minor season (Belg) not including the current season<br>**How much?**<br>**1 = All or a lot**<br>**2 = Some**<br>**3 = A bit**<br>**4 = None**<br>**98 = Don't know**		
				Sold<br><br>B	Consumed<br><br>C	Other use<br><br>D
Staples	502	Maize	|__|	|__||__|	|__||__|	|__||__|
	503	Teff	|__|	|__||__|	|__||__|	|__||__|
	504	Wheat	|__|	|__||__|	|__||__|	|__||__|
	505	Barley	|__|	|__||__|	|__||__|	|__||__|
	506	Sorghum	|__|	|__||__|	|__||__|	|__||__|
	507	Millet	|__|	|__||__|	|__||__|	|__||__|
	508	Rice	|__|	|__||__|	|__||__|	|__||__|
	509	Oat	|__|	|__||__|	|__||__|	|__||__|
	510	Other cereals	|__|	|__||__|	|__||__|	|__||__|
Pulses (legumes)	511	Bean	|__|	|__||__|	|__||__|	|__||__|
	512	Haricot bean	|__|	|__||__|	|__||__|	|__||__|
	513	Lentil (Miser)	|__|	|__||__|	|__||__|	|__||__|
	514	Grass pea (guaya)	|__|	|__||__|	|__||__|	|__||__|
	515	Chickpea	|__|	|__||__|	|__||__|	|__||__|
	516	Field pea (Ater)	|__|	|__||__|	|__||__|	|__||__|
	517	Soya bean	|__|	|__||__|	|__||__|	|__||__|
	518	Other legumes	|__|	|__||__|	|__||__|	|__||__|
Oil Crops	519	Niger seed (Nug)	|__|	|__||__|	|__||__|	|__||__|
	520	Sunflower	|__|	|__||__|	|__||__|	|__||__|
	521	Sesame	|__|	|__||__|	|__||__|	|__||__|
	522	Linseed	|__|	|__||__|	|__||__|	|__||__|
	523	Rapeseed (Gomenzer)	|__|	|__||__|	|__||__|	|__||__|
	524	Lupine	|__|	|__||__|	|__||__|	|__||__|
	525	Nuts		|__||__|	|__||__|	|__||__|
	526	Other oil crops	|__|	|__||__|	|__||__|	|__||__|
Root crops/ tubers/ vegetables	527	Cassava	|__|	|__||__|	|__||__|	|__||__|
	528	Enset	|__|	|__||__|	|__||__|	|__||__|
	529	Irish potato	|__|	|__||__|	|__||__|	|__||__|
	530	Sweet potato	|__|	|__||__|	|__||__|	|__||__|
	531	Sweet potato - orange flesh	|__|	|__||__|	|__||__|	|__||__|

	532	Onion	|__|	|__||__|	|__||__|	|__||__|
	533	Pepper	|__|	|__||__|	|__||__|	|__||__|
	534	Tomato	|__|	|__||__|	|__||__|	|__||__|
	535	Cabbage	|__|	|__||__|	|__||__|	|__||__|
	536	Other light green leafy vegetables	|__|	|__||__|	|__||__|	|__||__|
	537	Kale	|__|	|__||__|	|__||__|	|__||__|
	538	Other dark green leafy vegetables	|__|	|__||__|	|__||__|	|__||__|
	539	Carrot	|__|	|__||__|	|__||__|	|__||__|
	540	Other roots or tubers	|__|	|__||__|	|__||__|	|__||__|
	541	Other vegetables	|__|	|__||__|	|__||__|	|__||__|
Perennial crops/ fruits	542	Coffee	|__|	|__||__|	|__||__|	|__||__|
	543	Chat (khat)	|__|	|__||__|	|__||__|	|__||__|
	544	Banana	|__|	|__||__|	|__||__|	|__||__|
	545	Orange	|__|	|__||__|	|__||__|	|__||__|
	546	Mango	|__|	|__||__|	|__||__|	|__||__|
	547	Hop (Gesho)	|__|	|__||__|	|__||__|	|__||__|
	548	Avocado	|__|	|__||__|	|__||__|	|__||__|
	549	Lemon	|__|	|__||__|	|__||__|	|__||__|
	550	Papaya	|__|	|__||__|	|__||__|	|__||__|
	551	Guava	|__|	|__||__|	|__||__|	|__||__|
	552	Water Melon	|__|	|__||__|	|__||__|	|__||__|
	553	Tirngo fruit	|__|	|__||__|	|__||__|	|__||__|
	554	Other perennial crops	|__|	|__||__|	|__||__|	|__||__|
	555	Other fruits	|__|	|__||__|	|__||__|	|__||__|

556	Does this household own any livestock, herds, other farm animals, or poultry?	**1 = Yes**<br>**0 = No → GO to 577**	|__|

How many of the following animals does this household own?	**For each: Enter number. If none, enter 0**		
	557	Chickens	|__|__|__|
	558	Goats	|__|__|__|
	559	Sheep	|__|__|__|
	560	Donkeys	|__|__|__|
	561	Horses	|__|__|__|
	562	Mules	|__|__|__|
	563	Camels	|__|__|__|
	564	Milk cows	|__|__|__|
	565	Oxen	|__|__|__|

In the past 2 growing major (Meher) seasons and minor (Belg) growing seasons, not including the current season, please describe all animal source foods (meat, eggs, milk, dairy, fish, other) that you have produced on your household farm in the same period.

→Then ask how much was sold, consumed or used for another purpose.

Group		Animal source food (unit)	During the previous Major seasons (Meher) and Minor season (Belg) not including the current season			
			Does HH produce? **1=yes 0=no** *(If no, skip to the next item)* A	How much? **1 = All or a lot 2 = Some 3 = A bit 4 = None 98 = Don't know**		
				Sold B	Consumed C	Storage, losses, animal feed or other us D
All	566	Chicken eggs	|__|	|__||__|	|__||__|	|__||__|
	567	Chicken meat	|__|	|__||__|	|__||__|	|__||__|
	568	Goat milk	|__|	|__||__|	|__||__|	|__||__|
	569	Goat meat	|__|	|__||__|	|__||__|	|__||__|
	570	Camel milk	|__|	|__||__|	|__||__|	|__||__|
	571	Sheep meat	|__|	|__||__|	|__||__|	|__||__|
	572	Cow milk	|__|	|__||__|	|__||__|	|__||__|
	573	Cow other dairy	|__|	|__||__|	|__||__|	|__||__|
	574	Beef	|__|	|__||__|	|__||__|	|__||__|
	575	Other meat (e.g. wild animals)	|__|	|__||__|	|__||__|	|__||__|
	576	Farmed fish	|__|	|__||__|	|__||__|	|__||__|

At any time during the previous Major seasons (Meher) and Minor season (Belg) not including the current season, **Did you:**	577	Use improved seed varieties for any of your crops?	**1 = Yes 0 = No 98 = Don't know**	|___|___|
	578	Keep improved varieties of livestock?	**1 = Yes 0 = No 98 = Don't know**	|___|___|
	579	Use animal manure to fertilise your crops?	**1 = Yes 0 = No 98 = Don't know**	|___|___|
	580	Use any other source of fertiliser on your crops?	**1 = Yes 0 = No 98 = Don't know**	|___|___|
	581	Irrigate your crops?	**1 = Yes 0 = No 98 = Don't know**	|___|___|
	582	Rotate your crops from one field to another when planting?	**1 = Yes 0 = No 98 = Don't know**	|___|___|
	583	Harvest water during the rains?	**1 = Yes 0 = No 98 = Don't know**	|___|___|
	584	Practice intercropping?	**1 = Yes 0 = No 98 = Don't know**	|___|___|

585	Have you ever taken any steps to reduce soil erosion on your farm?	**1 = Yes 0 = No → Go to 591 98 = Don't know → Go to 591**	|___|___|

What steps did you take to reduce soil erosion?	**For each mentioned: 1=Yes 0=No**		
	586	Plant trees or shrubs	|__|
	587	Terracing	|__|
	588	Use drainage system	|__|
	589	Other	|__|

590	Have you received any inputs for your farm from a social/government programme?	**1 = Yes**<br>**0 = No → Go to 600**	|__|

What farm inputs have you received?<br><br>**READ THE LIST.**	**For each mentioned: 1=Yes 0=No**		
	591	Seeds	|__|
	592	Improved seeds	|__|
	593	Livestock or poultry	|__|
	594	Improved varieties of livestock/ poultry	|__|
	595	Aquaculture (fish)	|__|
	596	Fertiliser	|__|
	597	Irrigation equipment or support	|__|
	598	Farm equipment	|__|
	599	Other	|__|

Module 6 – Water, sanitation and hygiene

ABOUT THE HOUSEHOLD

600	What is the main source of drinking water for the household?<br><br>Do not read list	**1 = Piped connection into house**<br>**2 = Piped connection into yard**<br>**3 = Public standpipes**<br>**4 = Boreholes**<br>**5 = Protected dug wells**<br>**6 = Protected springs**<br>**7 = Rainwater collection**<br>**8 = Surface water**<br>**9 = Open dug wells**<br>**10 = Unprotected springs**<br>**11 = Vendor provided water**<br>**12 = Bottled water**<br>**13 = Tanker**	|___|___|
601	Do you do anything to your household water to make it safer to drink?	**1 = Yes**<br>**0 = No → GO to 603**<br>**98 = Don't know → GO to 603**	|___|___|
602	If yes, what is the main thing you do?	**1 = Let it stand and settle**<br>**2 = Strain through a cloth**<br>**3 = Use water filter (ceramic/sand/composite/etc)**<br>**4 = Boil**<br>**5 = Solar disinfection**<br>**6 = Add bleach/chlorine**<br>**99 = Other**<br>**98 = Don't know**	|___|___|
603	What is the usual place of defecation for family members?	**1 = No facility/bush/field**<br>**2 = Pit toilet/latrine used by this household only**<br>**3 = Toilet/latrine shared with other households**	|__|
604	Please show us the toilet or pit latrine that your family usually use.	**1= Observed**<br>**2= Not observed – not in dwelling/yard/plot → GO to 606**<br>**3 = Not observed – no permission to see → GO to 606**	|__|

605	OBSERVATION ONLY<br><br>OBSERVE THE TYPE OF LATRINE/TOILET	**1 = Basic pit toilet/latrine**<br>**2 = Improved pit toilet/latrine**<br>**3 = Composting**<br>**4 = Flush or pour-flush toilet to a pit or septic tank**	|__|
606	How does your HH primarily dispose of HH waste?	**1 = Collected by municipality**<br>**2 = Buried**<br>**3 = Collected by private establishment**<br>**4 = Dumped in street/open space**<br>**5 = Disposed in the compound**<br>**6 = Dumped in river**<br>**7 = Burned**<br>**8 = Other**	|__|
607	Please show me where members of your household most often wash their hands.	**1= Observed**<br>**2= Not observed – not in dwelling/yard/plot → GO to 610**<br>**3 = Not observed – no permission to see → GO to 610**	|__|
608	OBSERVATION ONLY:<br><br>OBSERVE PRESENCE OF WATER AT THE SPECIFIC PLACE FOR HANDWASHING.	**1 = Water is available**<br>**2 = Water is not available**	|__|
609	OBSERVATION ONLY:<br>OBSERVE PRESENCE OF<br><br>(SEE LIST)	**1 = Soap or detergent (bar, liquid, powder, paste)**<br>**2 = Ash, mud, sand**<br>**3 = None**	|__|
610	Do you have a confined space (beret/gata) to keep livestock?	**1 = Yes**<br>**0 = No**<br>**3 = Do not have livestock**	|__|
611	Do you keep poultry in cages/confined systems (kote)?	**1 = Yes**<br>**0 = No**<br>**3= Do not have poultry**	|__|

What do you think are the activities before which you should wash your hands with soap?<br><br>DO NOT PROMPT.	**For each mentioned: 1=Yes 0=No**		
	612	Before preparing food	|__|
	613	Before touching or eating food	|__|
	614	Before feeding a child or other person	|__|
	615	Praying	|__|
	616	Don't know	|__|
What do you think are the activities after which you should wash your hands with soap?<br><br>DO NOT PROMPT.	**For each mentioned: 1=Yes 0=No**		
	617	After defecation or urinating	|__|
	618	After handling animals and their waste	|__|
	619	After house work or field work	|__|
	620	After touching pets or handling animals and their waste	|__|
	621	After blowing nose or coughing	|__|
	622	After cleaning a child's bottom	|__|
	623	None	|__|
	**For each mentioned: 1=Yes 0=No**		
	624	To keep out of house	|__|
	625	To keep away from water source	|__|

What do you think are the reasons to keep poultry and livestock in a confined space?<br><br>DO NOT PROMPT.	626	To reduce infectious disease	|__|
	627	To protect livestock/poultry	|__|
	628	Other	|__|

Module 7 – Household Characteristics

ABOUT THE HOUSEHOLD			
700	*First ask who is the head of the household.*<br><br>What is the religion of the head of household?	**1 = Orthodox<br>2 = Catholic<br>3 = Protestant<br>4 = Muslim<br>5 = Other<br>6 = No religion**	|__|
701	What is the ethnic group of the household head?	**1 = Agew<br>2 = Amhara<br>3 = Gamo<br>4 = Gofa<br>5 = Sidama<br>6 = Gedeo<br>7 = Gurage<br>8 = Hadiya<br>9 = Tembaro<br>10= Kembata<br>11= Oromo<br>12 = Silite<br>13 = Tigray<br>14 = Welayita<br>15 = Other Ethiopian National Groups**	|___|___|
702	Do you own this house?	**1 = Yes<br>0 = No**	|__|
703	What is the main material of the walls?<br><br>Observe	**1 = No walls<br>2 = Natural materials (cane, wood, mud, straw)<br>3 = Stone with mud<br>4 = Stone/bricks with cement<br>98 = Other**	|___|___|
704	What is the main floor material?<br><br>Observe	**1 = Natural floor (earth/sand/dung)<br>2 = Rudimentary floor (wood/palm/bamboo)<br>3 = Finished floor (polished wood/ vinyl/tiles/cement/carpet)<br>98 = Other**	|___|___|
705	What is the main material of the roof?<br><br>Observe	**1 = Thatch/grass or leaves<br>2 = Iron sheets or tiles<br>98 = Other**	|___|___|
706	What type of fuel does your household mostly use for cooking?<br><br>**Do not read list**	**1 = Dung<br>2 = Firewood/straw<br>3 = Charcoal<br>4 = Kerosene<br>5 = Gas (methane/biogas)<br>6 = Electricity<br>98 = Other**	|___|___|
707	Is the house connected to electricity?	**1 = Yes<br>0 = No**	|__|

	Enter number of items (zero if none)		
In total, how many of the following items are owned by residents of this household?<br><br>**Add the household total for each item**	708	A kerosene lamp/pressure lamp	|__||__|
	709	Mobile pone	|__||__|
	710	Cart/	|__||__|
	711	Bicycle	|__||__|
	712	Motorcycle	|__||__|
	713	Radio	|__||__|
	714	Television	|__||__|
	715	Car/tractor/bajaj	|__||__|

Module 8 – Anthropometry and Bloods

**FOR CHILDREN 6-47 MONTHS**<br>**Note: Take the anthropometric measurements at the end of the interview to not interrupt the mother.**			
800	Presence of Bilateral Oedema	**1 = Yes**<br>**0 = No → GO to 284**	|__|
801	If yes for presence of bilateral oedema, refer the child's caregiver to health post for treatment of the child.<br><br>**Have you referred the caregiver and answered any questions?**	**1 = Yes**<br>**0 = No**	|__|
802	Mid Upper Arm Circumference (MUAC) in millimetres<br><br>***Take two <u>separate</u> MUAC measures to the nearest millimetre.***	☐☐☐ mm	
803		☐☐☐ mm	
804	Child's Weight in kilograms<br><br>Take if the child is able and willing to stand on the scale alone<br>➔ If not, skip to 807<br><br>***Take two <u>separate</u> weight measures to the nearest tenth of a kilogram.***<br><br>***If the two measurements are not within 0.1kg, take a separate third weight measure and record.***	☐☐.☐ kg	
805		☐☐.☐ kg	
806		☐☐.☐ kg	

807	Mother's Weight in kilograms<br><br>Take if the child is NOT able and willing to stand on the scale alone<br><br>***Take two <u>separate</u> weight measures to the nearest tenth of a kilogram.***<br><br>***If the two measurements are not within 0.1kg, take a separate third weight measure and record.***	☐☐.☐ kg
808		☐☐.☐ kg
809		☐☐.☐ kg
810	Mother and child's weight in kilograms<br><br>Take if the child is NOT able and willing to stand on the scale alone<br><br>***Take two <u>separate</u> weight measures to the nearest tenth of a kilogram.***<br><br>***If the two measurements are not within 0.1kg, take a separate third weight measure and record.***	☐☐.☐ kg
811		☐☐.☐ kg
812		☐☐.☐ kg
813	Height/Length in centimetres<br><br>***Take two <u>separate</u> height/length measures to the nearest tenth of a centimetre.***<br><br>***If the two measurements are not within 0.7cm, take a separate third height/length measure and record.***	☐☐☐.☐ cm
814		☐☐☐.☐ cm
815		☐☐☐.☐ cm
816	**Measured lying down or standing up?**	**1 = Lying down**<br>**2 = Standing up**<br>**3 = Not measured** |__|

<u>FINGER PRICK CONSENT</u>

ASK CONSENT FOR ANAEMIA TEST FROM MOTHER/CARETAKER

As part of this survey, we are asking children to take an anaemia test. Anaemia is a serious health problem that usually results from poor nutrition, infection, or chronic disease. This survey will assist the government to develop services to prevent and treat undernutrition including anaemia.

We request that children give a few drops of blood from a finger. The equipment used in taking the blood is clean and completely safe. It has never been used before and will be thrown away after each test. The blood will be tested for anaemia immediately, and the result told to you right away. The result will be kept strictly confidential and will not be shared with anyone other than members of our survey team.

Do you have any questions?			
817	You can say yes to the test, or you can say no. It is up to you to decide.<br><br>Do you give your consent for (NAME) to participate in the anaemia test?	**1 = Yes**<br>**0 = No → Go to 300**	|__|
818	HAEMOGLOBIN	□□.□ g/DL	

<u>Thank you for your time</u>

<u>End of interview</u>
